# Adjusting total body iron for inflammation: Biomarkers Reflecting Inflammation and Nutritional Determinants of Anemia (BRINDA) project

**DOI:** 10.3945/ajcn.116.142307

**Published:** 2017-06-14

**Authors:** Zuguo Mei, Sorrel ML Namaste, Mary Serdula, Parminder S Suchdev, Fabian Rohner, Rafael Flores-Ayala, O Yaw Addo, Daniel J Raiten

**Affiliations:** 1 Nutrition Branch, CDC, Atlanta, GA;; 2 Strengthening Partnerships, Results, and Innovations in Nutrition Globally, Arlington, VA;; 3 Helen Keller International, Washington, DC;; 4 Emory University, Atlanta, GA;; 5 GroundWork, Fläsch, Switzerland; and; 6 *Eunice Kennedy Shriver* National Institute of Child Health and Human Development, NIH, Bethesda, MD

**Keywords:** acute-phase proteins, C-reactive protein, ferritin, inflammation, iron deficiency, preschool-age children, soluble transferrin receptor, total body iron, women of reproductive age, α-1-acid glycoprotein

## Abstract

**Background:** Total body iron (TBI) that is calculated from ferritin and soluble transferrin receptor (sTfR) allows for the evaluation of the full range of iron status from deficiency to excess. However, both ferritin and sTfR are affected by inflammation and malaria, which may require a statistical adjustment. TBI has been used to assess iron status in the United States, but its use worldwide and in settings with inflammation has been limited.

**Objective:** We examine whether inflammation-adjusted ferritin and sTfR concentrations affect TBI values and the prevalence of low TBI (<0 mg/kg) in preschool children (PSC) (age range: 6–59 mo) and women of reproductive age (WRA) (age range: 15–49 y).

**Design:** Cross-sectional data for PSC (8 surveys; *n* = 8413) and WRA (4 surveys; *n* = 4258) from the Biomarkers Reflecting the Inflammation and Nutritional Determinants of Anemia (BRINDA) project were analyzed individually and combined. TBI and the prevalence of low TBI were compared following 3 adjustment approaches for ferritin and sTfR: *1*) the exclusion of individuals with inflammation (C-reactive protein concentration >5 mg/L or α-1-acid glycoprotein concentration >1 g/L), *2*) the application of arithmetic correction factors, and *3*) the use of regression correction.

**Results:** Regardless of the method that was used to adjust ferritin and sTfR for inflammation, the adjusted mean TBI decreased in both PSC and WRA compared with unadjusted values. Subsequently, inflammation-adjusted TBI increased the prevalence of low TBI by a median of 4–14 percentage points (pps) in PSC and 1–3 pps in WRA compared with unadjusted TBI. The regression approach resulted in a greater median increase than was achieved with the exclusion or correction-factor approaches, and accounting for malaria in addition to inflammation did not have an added effect on the prevalence estimates.

**Conclusion:** The prevalence of low TBI is underestimated if it is not adjusted by inflammation, particularly in children living in areas with a high prevalence of inflammation.

## INTRODUCTION

Iron deficiency (ID) is thought to be the most-common known form of nutritional deficiency, particularly in preschool children (PSC) and women of reproductive age (WRA) (1–4). In 2004, the WHO and CDC recommended that measurements of ferritin and soluble transferrin receptor (sTfR) would provide the best approach for measuring the iron status of populations. The WHO and CDC also suggested that, if funding is available, it would be useful to measure one or both of the acute-phase proteins C-reactive protein (CRP) or α-1-acid glycoprotein (AGP) to account for inflammation in the interpretation of ferritin concentrations (5).

The interpretation of ferritin concentrations as a measure of low iron stores becomes difficult in areas with high inflammation or infection (6–8) because ferritin concentrations increase during inflammation. Ferritin is a positive acute-phase protein; thus, not accounting for inflammation can lead to a significant underestimation of the prevalence of low iron stores at the population level (8). In contrast with ferritin concentrations, sTfR is increased in ID, and the interpretation of sTfR concentrations has been thought to be only marginally influenced by the inflammatory response (9). However, studies have shown that sTfR values may be affected by physiologic factors that affect erythropoietic activity such as malaria and low-grade chronic inflammation that may limit the utility of sTfR as a specific marker of iron nutrition (10–12). sTfR concentrations are affected by inflammation; thus, not accounting for inflammation may lead to an overestimation of the prevalence of ID at the population level.

In 2003, Cook and colleagues (13, 14) introduced a method for estimating total body iron (TBI) on the basis of the ratio of sTfR-to-ferritin concentrations. This quantitative estimate, which expresses TBI on the basis of body weight, has been suggested to allow for an evaluation of the full range of iron status from deficiency to excess within a population. TBI has several advantages including that *1*) it yields a measure of the size of the iron deficit that is independent of the hemoglobin concentration, and *2*) it is the only method that is based on actual experimental observations (15). Furthermore, there is reasonably good agreement between the estimated prevalence of ID by TBI and the previously described ferritin model (measuring ferritin, transferrin saturation, and erythrocyte protoporphyrin) in PSC, WRA (16), and pregnant women (17).

Although successfully used in the United States to assess the prevalence of ID of PSC, WRA, and pregnant women (16, 17) and for monitoring purposes in the Healthy People 2020 objectives (18), TBI has been used less often in other parts of the world, particularly in areas with high levels of inflammation and infections including malaria. Because of the potential of TBI to provide a quantitative estimate of iron status in individuals with ID, a normal iron balance, and increased iron stores (16), it is important to examine whether TBI is affected by inflammation to determine its utility in such settings.

Both ferritin and sTfR are affected by inflammation (6, 12), but they affect the estimated prevalence of ID in different directions (increased estimates of ID with the use of inflammation-adjusted ferritin concentrations and decreased estimates of ID with the use of inflammation-adjusted sTfR concentrations). Because TBI is a calculated ratio from ferritin and sTfR, it can be hypothesized that TBI would not be affected by inflammation because the effects of inflammation might cancel each other out. In this article, we examined whether inflammation-adjusted ferritin and sTfR concentrations affect estimates of TBI and the prevalence of low TBI in PSC and WRA.

## METHODS

We used data from the Biomarkers Reflecting Inflammation and Nutritional Determinants of Anemia (BRINDA) project (www.BRINDA-nutrition.org) (19). The BRINDA protocol was reviewed by the institutional review boards of the NIH and was deemed non–human subjects research. The methods for identifying data sets, inclusion and exclusion criteria, and data management for the BRINDA project have been described in detail in the methodologic overview of this supplement, which is an open access publication (20). The surveys were nationally or regionally representative, and the data inclusion criteria were as follows: *1*) surveys conducted after 2004, *2*) target groups included PSC or nonpregnant WRA, and *3*) surveys measured ≥1 marker of iron status (ferritin or sTfR) or vitamin A status (retinol binding protein or retinol) and ≥1 marker of inflammation (AGP or CRP). Observations were included in this analysis if they had measures of both ferritin and sTfR plus inflammation (both CRP and AGP). Of the 16 PSC and 10 WRA BRINDA data sets, data from 8 PSC data sets and 4 WRA data sets were available for analysis for this article. Malaria was measured in 5 PSC and 3 WRA in these data sets.

### Laboratory analysis

Venous or capillary blood was collected from each respondent, and plasma or serum was stored at −20°C until analysis. Ferritin, sTfR, CRP, and AGP concentrations were assessed with the use of a sandwich ELISA at the VitMin Laboratory (21). Malaria was assessed with the use of microscopy [in Kenya and Côte d’Ivoire, (22)], the Paracheck Pf rapid diagnostic test (Orchid Biomedical System) in Liberia, and a histidine-rich protein 2 rapid diagnostic test (Cellabs Pty Ltd.) in Cameroon. Additional information on laboratory methods is further described in the methodologic overview in this supplement (20).

### Case definitions

TBI was calculated from sTfR and ferritin concentrations with the use of the following formula from Cook and colleagues (13, 14):


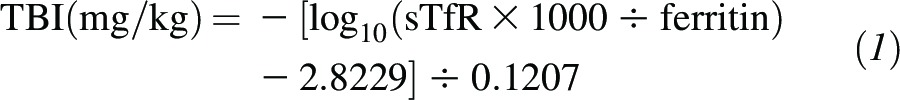


Positive TBI values represent existing storage iron, whereas negative TBI values (<0 mg/kg) indicate tissue ID with increasing severity of ID as the values of TBI further decrease (13, 14). Malaria was defined as a dichotomous variable (positive or negative). Inflammation was considered present if CRP concentrations were >5 mg/L or AGP concentrations were >1 g/L (8, 21).

### Statistical analysis

Descriptive statistics were calculated with the use of STATA 12.0 software (StataCorp) and cross-checked with SAS 9.4 software (SAS Institute). The Taylor linearization method was used to obtain unbiased estimates that incorporated the weight, strata, and cluster (as applicable) when analyzing individual countries. To combine data, individual survey analyses, with the complex survey design accounted for, were performed with the use of the “survey” package in R 3.2.2 software (R Core Team) (23). Then, individual survey estimates were combined with the use of a meta-analysis approach with the use of the metafor package in R 3.2.2 software (R Core Team) (24). The heterogeneity of estimates across the surveys was assessed with the use of Cochrane’s heterogeneity test.

Several approaches to adjust TBI for inflammation and malaria were explored as are described in detail in the BRINDA methods article (20). First, the prevalence of low TBI (<0 mg/kg) was calculated on the basis of unadjusted ferritin and sTfR estimates, which are referred to as unadjusted estimates. Subsequently, the following 3 adjustment approaches were applied to account for inflammation and malaria: *1*) the exclusion of subjects with any inflammation, *2*) correction factors (CFs), and *3*) regression corrections (RCs).

Because TBI is a ratio that is calculated from ferritin and sTfR, we used adjusted ferritin and sTfR concentrations before applying the TBI equation. On the basis of the results from previous BRINDA work (6, 12), a moderately positive association between ferritin with CRP and AGP was shown, and adjusting ferritin for both CRP and AGP was suggested. However, because sTfR was more affected by chronic inflammation, and elevated CRP prevents the rise of sTfR during the early acute-phase response, only adjusting sTfR for AGP was recommended (12). Thus in our analysis, we adjusted ferritin for both CRP and AGP but only adjusted AGP for sTfR.

### Exclusion approach

The exclusion approach excluded individuals with elevated CRP or AGP (defined as a CRP concentration >5 mg/L or AGP concentration >1 g/L) and calculated the prevalence of low TBI in the remaining individuals; this categorical approach resulted in a smaller sample size.

### CF approach

The CF approach, as proposed by Thurnham et al. (8), uses arithmetic CFs that are derived from the following 4-group inflammation-adjustment model: *1*) reference (both CRP concentration ≤5 mg/L and AGP concentration ≤1 g/L); *2*) incubation (both CRP concentration >5 mg/L and AGP concentration ≤1 g/L); *3*) early convalescence (both CRP concentration >5 mg/L and AGP concentration >1 g/L); and *4*) late convalescence (both CRP concentration ≤5 mg/L and concentration AGP >1 g/L). We also calculated CFs by grouping inflammation into 2 groups in which CRP and AGP were used independently. CFs were defined as the ratio of geometric means of the reference group (nonelevated CRP and AGP) to those of the respective inflammation groups. CFs were calculated with the use of internal survey-specific data [termed the internal correction factor (ICF)] and from BRINDA’s meta-analysis values [BRINDA correction factors (BCFs)].

### RC approach

The RC approach uses linear regression to adjust ferritin concentrations by the concentrations of CRP and AGP, sTfR concentrations by the concentration of AGP on a continuous scale, and malaria as a dichotomous variable. In brief, adjusted TBI was calculated with adjusted ferritin and adjusted sTfR concentrations as follows:


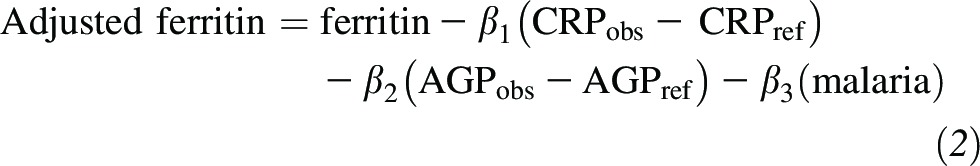



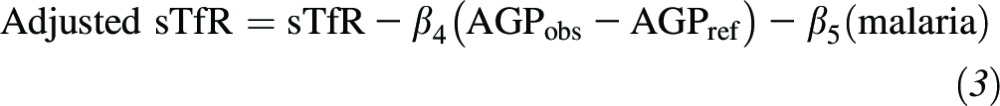


β_1_ is the CRP regression coefficient, β_2_ and β_4_ are the AGP regression coefficients, β_3_ and β_5_ are the malaria regression coefficients, obs is the observed value, and ref is the external reference value generated to define low inflammation [maximum value of the lowest AGP decile with the use of combined BRINDA data with non-logged reference values (AGP in PSC: 0.59 g/L; AGP in WRA: 0.54 g/L]. The correction was only applied to individuals with either ln CRP greater than ln CRP_ref_, ln AGP greater than ln AGP_ref_, or both to avoid overadjustments (20). An illustrative example of the use of the RC approach to adjust ferritin and sTfR for inflammation in PSC in Liberia is provided in **Supplemental Figure 1**.

The RC approach is presented based on each individual survey [internal regression correction (IRC)] or with the use of slope estimates from a BRINDA meta-analysis [BRINDA regression correction (BRC)]. The BRC approach entailed replacing the CRP and AGP β coefficients in the adjusted ferritin Equation *2* and the AGP β coefficients in the adjusted sTfR equation *3* with the meta-analysis β coefficients. The same external reference value was used when applying both the IRC and BRC approaches.

### Comparing adjustments

Unadjusted and adjusted prevalence estimates of low TBI were compared with the use of McNemar’s chi-square test; statistical significance was defined as *P* < 0.05 before applying the Bonferroni corrections to correct for multiple comparisons (*P* = 0.05 ÷ *k*, where *k* equals the number of comparisons).

## RESULTS

### Participant characteristics

Our study sample was restricted to participants with no missing values for ferritin, sTfR, CRP, AGP, or malaria (in countries that measured malaria), which resulted in a total of 8413 PSC and 4258 WRA observations. Participants who were excluded because of missing ferritin, sTfR, CRP, AGP, or malaria data did not differ from those who were included with regard to sex, age, or socioeconomic status (data not shown). PSC had variability in the minimum and maximum age with an age range of 6–59 mo, whereas all WRA had an age range of 15–49 y (**Table 1**). The prevalence of inflammation varied across surveys in PSC (CRP concentration >5 mg/L: 13.9–40.4%; AGP concentration >1 g/L: 21.2–64.5%) and WRA (CRP concentration >5 mg/L: 7.9–19.7%; AGP concentration >1 g/L: 7.2–26.9%) (Table 1). The prevalence of malaria varied by 13 percentage points (pps) in both PSC (19.7–32.5%) and WRA (5.0–17.9%) (Table 1).

**TABLE 1:** Characteristics in preschool children and women of reproductive age: the BRINDA project^1^

			% (95% CI)			
Country	*n*	Age^2^	CRP concentration >5 mg/L	AGP concentration >1 g/L	CRP concentration >5 mg/L or AGP concentration >1 g/L	Malaria positive
Preschool children						
Bangladesh	1493	8.3 (6–11)	14.3 (11.8, 16.7)	33.4 (29.9, 36.9)	35.8 (32.2, 39.5)	—
Cameroon	774	30.8 (12–59)	37.5 (32.7, 42.3)	39.3 (33.7, 45.0)	48.3 (43.1, 53.5)	25.9 (20.2, 31.5)
Côte d’Ivoire	733	31.7 (6–59)	40.4 (36.5, 44.3)	64.5 (60.3, 68.6)	67.5 (63.8, 71.3)	27.2 (22.3, 32.0)
Kenya 2007	888	19.9 (6–35)	27.8 (23.9, 31.7)	64.2 (60.2, 68.2)	66.0 (61.9, 70.1)	19.7 (15.8, 23.6)
Kenya 2010	843	21.4 (6–35)	34.2 (29.6, 38.7)	60.7 (56.0, 65.4)	61.9 (57.2, 66.6)	32.5 (28.4, 36.6)
Laos	481	33.1 (6–59)	16.6 (11.2, 22.1)	41.7 (34.0, 49.4)	44.0 (36.6, 51.5)	—
Liberia	1434	19.9 (6–35)	29.5 (26.5, 32.5)	56.2 (52.5, 60.0)	59.1 (55.6, 62.7)	29.4 (26.2, 32.6)
Philippines	1767	15.0 (6–23)	13.9 (11.6, 16.2)	21.2 (17.7, 24.6)	26.0 (22.4, 29.5)	—
Women of reproductive age						
Cameroon	751	27.2 (15–49)	17.8 (14.8, 20.7)	7.2 (5.1, 9.3)	19.7 (16.6, 22.9)	15.0 (11.3, 18.6)
Côte d’Ivoire	816	27.6 (15–49)	19.7 (16.5, 22.8)	26.9 (23.5, 30.4)	33.7 (29.6, 37.9)	5.0 (3.4, 6.5)
Laos	816	29.3 (15–49)	7.9 (5.6, 10.2)	9.3 (7.1, 11.6)	13.9 (10.9, 16.8)	—
Liberia	1875	28.6 (15–49)	14.3 (12.1, 16.4)	10.4 (8.7, 12.2)	18.5 (16.2, 20.8)	17.9 (15.3, 20.4)

1 Countries are ordered alphabetically. AGP, α-1-acid-glycoprotein; BRINDA, Biomarkers Reflecting Inflammation and Nutritional Determinants of Anemia; CRP, C-reactive protein.

2 All values are means (minimums to maximums). Age is given in months in children and years in women of reproductive age.

### Stratified analysis by CRP or AGP status

A stratified analysis by CRP status (≤5 compared with >5 mg/L) showed that the prevalence of low TBI in the >5-mg/L group was significantly (*P* < 0.05) lower than in the ≤5-mg/L group in all the 8 surveys in PSC and in 3 of 4 surveys in WRA (**Supplemental Table 1**). A stratified analysis by AGP status (≤1 compared with >1 g/L) showed that the prevalence of low TBI in the ≤1-g/L group was significantly higher than in the >1-g/L group in 7 of 8 surveys in PSC but in only 1 of 4 surveys in WRA (**Supplemental Table 2**). In the 5 surveys that measured malaria in PSC and 3 surveys that measured malaria in WRA, the prevalence of low TBI in the malaria-negative group was significantly higher than in the malaria-positive group in 4 of 5 surveys in PSC but not in the 3 surveys in WRA (**Supplemental Table 3**).

### Mean TBI and prevalence of low TBI

The unadjusted mean TBI ranged from 1.4 to 5.5 mg/kg in PSC and from 3.8 to 6.8 mg/kg in WRA (**Table 2**). However, regardless of the method that was used to adjust inflammation for ferritin and sTfR, the inflammation-adjusted mean TBI was decreased in both PSC and WRA compared with unadjusted values. In general, the TBI after RC adjustment had the lowest means compared with the means after CF adjustment and the unadjusted means (Table 2).

**TABLE 2:** Inflammation-adjusted mean total body iron in preschool children and women of reproductive age: the BRINDA project^1^

		Means (95% CIs), mg/kg						
Country	*n*	Unadjusted	Excluded subjects with CRP concentrations >5 mg/L or AGP concentrations >1 g/L^2^	Internal correction factor	BRINDA correction factor	Internal regression correction	BRINDA regression correction	Internal regression correction and malaria^3^
Preschool children								
Bangladesh	1493	3.3 (3.0, 3.6)	2.8 (2.4, 3.1)	2.8 (2.5, 3.1)	2.7 (2.4, 3.0)	2.1 (1.9, 2.4)	1.2 (1.0, 1.5)	—
Cameroon	774	2.5 (2.0, 2.9)	1.6 (1.0, 2.1)	1.5 (1.1, 1.9)	1.4 (1.0, 1.7)	0.4 (0.0, 0.8)	0.0 (−0.4, 0.4)	0.5 (0.1, 0.9)
Côte d’Ivoire	733	5.4 (5.0, 5.9)	4.4 (3.9, 5.0)	4.4 (4.0, 4.8)	4.0 (3.6, 4.4)	2.3 (1.9, 2.7)	2.4 (2.0, 2.8)	2.4 (2.1, 2.8)
Kenya 2007	888	1.4 (1.0, 1.8)	−0.2 (−0.7, 0.4)	−0.2 (−0.6, 0.2)	0.2 (−0.2, 0.6)	−1.6 (−2.0, −1.2)	−1.5 (−1.8, −1.1)	−1.5 (−1.9, −1.2)
Kenya 2010	843	2.0 (1.6, 2.3)	0.1 (−0.4, 0.6)	0.1 (−0.2, 0.4)	0.7 (0.4, 1.0)	−1.1 (−1.4, −0.8)	−0.8 (−1.1, −0.5)	−1.1 (−1.4, −0.8)
Laos	481	5.5 (4.9, 6.1)	5.1 (4.5, 5.7)	5.1 (4.5, 5.6)	4.8 (4.2, 5.3)	4.4 (3.9, 5.0)	3.4 (2.9, 4.0)	—
Liberia	1434	1.8 (1.5, 2.1)	0.7 (0.3, 1.0)	0.7 (0.4, 0.9)	0.7 (0.4, 0.9)	−1.1 (−1.4, −0.8)	−0.9 (−1.2, −0.6)	−1.0 (−1.3, −0.8)
Philippines	1767	2.9 (2.6, 3.2)	2.3 (2.0, 2.7)	2.3 (2.0, 2.7)	2.4 (2.1, 2.7)	1.9 (1.6, 2.2)	1.1 (0.7, 1.4)	—
Women of reproductive age								
Cameroon	751	4.0 (3.7, 4.3)	3.7 (3.4, 4.0)	3.7 (3.4, 4.0)	3.7 (3.4, 4.0)	3.4 (3.1, 3.7)	2.3 (1.9, 2.6)	3.4 (3.1, 3.7)
Côte d’Ivoire	816	4.7 (4.4, 5.0)	4.4 (4.0, 4.7)	4.4 (4.0, 4.7)	4.3 (4.0, 4.7)	3.5 (3.2, 3.8)	3.0 (2.7, 3.3)	3.6 (3.3, 3.9)
Laos	816	6.8 (5.9, 7.7)	6.7 (5.8, 7.5)	6.7 (5.8, 7.5)	6.7 (5.8, 7.5)	6.5 (5.7, 7.3)	5.5 (4.7, 6.4)	—
Liberia	1875	3.8 (3.5, 4.1)	3.6 (3.3, 3.8)	3.6 (3.3, 3.8)	3.6 (3.3, 3.8)	2.7 (2.4, 2.9)	2.3 (2.0, 2.5)	2.7 (2.5, 3.0)

1 AGP, α-1-acid-glycoprotein; BRINDA, Biomarkers Reflecting Inflammation and Nutritional Determinants of Anemia; CRP, C-reactive protein; sTfR, soluble transferrin receptor.

2 Effective sample sizes that are lower than stated *n*s as estimates were based on an uninflamed subpopulation.

3 On the basis of AGP and malaria adjustments to sTfR and CRP and AGP adjustments to ferritin.

There was variation in the unadjusted prevalence of low TBI (<0 mg/kg) across surveys. The prevalence ranged from 11.0% to 37.8% in PSC and from 11.4% to 15.1% in WRA (**Table 3**). In the surveys with both PSC and WRA data, the unadjusted prevalence of low TBI was similar between the 2 population groups in Côte d’Ivoire and Laos and was substantially lower in WRA in Cameroon and Liberia (Table 3).

**TABLE 3:** Inflammation-adjusted prevalence of total body iron (<0 mg/kg) in preschool children and women of reproductive age: the BRINDA project^1^

		% (95% CI)						
Country	*n*	Unadjusted	Excluded subjects with CRP concentrations >5 mg/L or AGP concentrations >1 g/L	Internal correction factor	BRINDA correction factor	Internal regression correction	BRINDA regression correction	Internal regression correction and malaria^2^
Preschool children								
Bangladesh	1493	15.7 (12.9, 18.6)	19.1 (15.2, 23.0)	18.3 (15.3, 21.2)	19.5 (16.9, 22.1)	23.6 (20.7, 26.5)	28.1 (25.3, 30.8)	—
Cameroon	774	23.9 (20.3, 27.5)	30.0 (24.6, 35.4)	28.5 (24.9, 32.2)	29.6 (25.9, 33.3)	37.7 (33.7, 41.7)	37.1 (33.2, 40.9)	36.3 (32.6, 40.0)
Côte d’Ivoire	733	11.3 (9.0, 13.6)	13.0 (8.2, 17.9)	13.4 (10.6, 16.1)	15.9 (13.0, 18.8)	26.2 (22.6, 29.8)	22.3 (19.0, 25.6)	26.0 (22.4, 29.6)
Kenya 2007	888	37.8 (34.0, 41.7)	50.7 (45.3, 56.0)	48.6 (44.5, 52.8)	46.2 (42.2, 50.2)	62.5 (58.8, 66.2)	54.8 (51.0, 58.7)	61.9 (58.2, 65.7)
Kenya 2010	843	31.4 (28.2, 34.7)	44.2 (37.6, 50.9)	44.8 (41.7, 48.0)	39.1 (35.9, 42.4)	56.7 (53.7, 59.7)	48.5 (45.4, 51.6)	56.5 (53.4, 59.6)
Laos	481	11.0 (7.6, 14.5)	13.8 (9.0, 18.7)	12.5 (8.8, 16.3)	14.6 (10.8, 18.5)	15.1 (11.2, 19.0)	16.3 (12.6, 20.1)	—
Liberia	1434	32.3 (29.0, 35.6)	42.0 (37.6, 46.5)	41.2 (37.6, 44.7)	40.0 (36.6, 43.5)	58.2 (54.5, 62.0)	50.6 (46.8, 54.4)	58.5 (54.7, 62.3)
Philippines	1767	21.3 (18.1, 24.6)	24.7 (20.6, 28.7)	23.7 (20.3, 27.2)	23.2 (19.8, 26.6)	26.5 (22.8, 30.2)	29.8 (26.3, 33.4)	—
Women of reproductive age								
Cameroon	751	11.6 (9.1, 14.2)	12.6 (9.8, 15.4)	12.6 (10.0, 15.2)	12.6 (10.0, 15.2)	12.9 (10.3, 15.6)	13.9 (11.1, 16.8)	12.9 (10.2, 15.5)
Côte d’Ivoire	816	11.4 (8.5, 14.3)	14.1 (10.1, 18.0)	12.8 (9.9, 15.8)	13.0 (10.2, 15.9)	15.5 (12.7, 18.3)	14.9 (12.0, 17.8)	15.5 (12.8, 18.2)
Laos	816	12.4 (8.7, 16.2)	13.5 (9.2, 17.7)	12.6 (8.9, 16.4)	13.0 (9.2, 16.7)	13.3 (9.2, 17.5)	13.9 (9.6, 18.2)	—
Liberia	1875	15.1 (13.1, 17.2)	16.2 (14.0, 18.3)	16.2 (13.9, 18.4)	16.2 (14.0, 18.4)	20.7 (17.7, 23.6)	19.1 (16.4, 21.7)	20.3 (17.6, 23.1)

1 AGP, α-1-acid-glycoprotein; BRINDA, Biomarkers Reflecting Inflammation and Nutritional Determinants of Anemia; CRP, C-reactive protein; sTfR, soluble transferrin receptor.

2 On the basis of AGP and malaria adjustments to sTfR and CRP and AGP adjustments to ferritin.

### Comparing prevalence of low TBI with the use of different approaches to adjust ferritin and sTfR for inflammation

In PSC, regardless of the adjustment method, the inflammation-adjusted prevalence of low TBI increased for all surveys compared with the unadjusted prevalence. The difference was more marked with the use of a continuous RC than with a categorical approach (exclusion or correction factor) (Table 3). As expected because of the similarity between approaches, the estimated prevalence of low TBI was comparable between the exclusion of individuals with inflammation and ICF approaches (sTfR was not adjusted for CRP in the latter approach). With the use of the exclusion approach, the estimated prevalence of low TBI resulted in an absolute median increase of 4.8 pps (range: 1.7–12.9 pps) compared with unadjusted values in PSC. With the CF approaches, the estimated prevalence of low TBI resulted in an absolute median increase of 3.6 pps (range: 1.5–13.4 pps) with the use of ICF and of 5.2 pps (range: 1.9–8.4 pp) with the use of BCF compared with unadjusted values in PSC (Table 3). However, the prevalence of low TBI with RC approaches resulted in an absolute median increase of 14.4 pps (range: 4.1–25.9 pps) with the use of IRC and of 12.8 pps (range: 5.3–18.3 pp) with the use of BRC compared with unadjusted values in PSC (Table 3).

In WRA, the differences between unadjusted low TBI and adjusted prevalences were much smaller than with PSC. In general, the use of RC approaches resulted in a higher prevalence of low TBI than the use of exclusion or CF approaches in WRA. The estimated prevalence of low TBI in WRA showed an absolute median increase of 1.1 pps (range: 1.0–2.7 pps) with the use of the exclusion approach, 1.1 pps (range: 0.2–1.4 pps) with the use of the ICF approach, 1.1 pps (range: 0.6–1.6 pps) with the use of BCF approach, 2.7 pps (range: 0.9–5.6 pps) with the use of IRC approach, and 2.9 pps (range: 1.5–3.5 pps) with the use of BRC compared with unadjusted values (Table 3).

### Comparing prevalence of low TBI with the use of different approaches to adjust TBI for inflammation and malaria

Adjustment for inflammation alone with the use of IRC approach (Table 2) resulted in similar point estimates of low TBI as with adjustment for inflammation plus malaria in PSC (NS in 4 surveys; *P* > 0.1) and WRA (NS in all 4 surveys) (Table 3).

## DISCUSSION

In this analysis, we examined whether inflammation-adjusted ferritin and sTfR concentrations affect TBI values and the prevalence of low TBI in >8000 PSC and >4000 WRA with the use of multicountry data that were regionally or nationally representative and contained both CRP and AGP to assess inflammation. We also examined whether inflammation and malaria need to be adjusted for in areas of varying prevalence of malaria and nonspecific inflammation when assessing the prevalence of low TBI. Associated analyses from the BRINDA project that are reported in this supplement confirm that inflammation-adjusted ferritin concentrations increase estimates of depleted iron stores, and inflammation-adjusted sTfR concentrations decrease estimates of iron-deficient erythropoiesis (6, 12). Our results suggest that TBI, which is calculated from ferritin and sTfR, is still affected by inflammation, and the effects of inflammation on ferritin and sTfR do not cancel each other out. Adjustment of TBI for both CRP and AGP led to a median increase in the estimated prevalence of low TBI ranging from 1.5 to 25.9 and 0.2 to 5.6 pps depending on the approach in PSC and WRA, respectively. Inflammation and malaria adjustments resulted in a similar prevalence of low TBI to that of inflammation alone. Thus, when both AGP and CRP are measured, there appears to be limited utility in adjusting TBI for malaria status.

The application of different adjustment approaches resulted in a high degree of variability in the adjusted prevalence of low TBI. As discussed in the BRINDA ferritin (6) and sTfR (12) articles on inflammation adjustment, each approach has advantages and disadvantages. The exclusion approach resulted in a loss of precision because of diminishing sample sizes and may have introduced bias. ICFs have the advantage of using the underlying inflammation profile on the basis of different stages of inflammation to adjust micronutrient biomarker concentrations. However, the precision is reduced compared with that of TCFs or BCFs because the proportion of the population with elevated inflammation is based on the sample size of the reference group. The positive relation of TBI with inflammation across the entire range of AGP and CRP concentrations that were observed in this large cross-country data set indicates that the categorizations that are used in the CF approach may underadjust for inflammation. In contrast, the RC approach uses linear regression to adjust ferritin and sTfR by the concentration of CRP and/or AGP on a continuous scale but requires advanced analytic skills.

The large sample size from representative surveys from multiple geographic areas and the comparability of laboratory methods are major strengths of this study. The main limitations are that the data we examined were all from cross-sectional surveys, the selection of data sets were based on convenience (i.e., the availability of data from BRINDA partners), and there was not a gold-standard measure of iron status to compare against. Longitudinal data could provide richer information to disentangle nutritional ID from the influence of other physiologic processes and examine changes in TBI, CRP, and AGP in response to an intervention. The use of CRP and AGP may incompletely capture inflammation, and thus, there may still be some bias when adjusting TBI. Another limitation of TBI is the lack of a standard sTfR-assay method and a standard reference material (5). However, efforts are underway to develop and characterize a serum that is based on WHO international reference material for sTfR assays.

TBI requires measurements of both ferritin and sTfR concentrations. In resource-limited settings, it is advantageous to measure as few biomarkers as necessary. Thus, if only one of the iron biomarkers (ferritin or sTfR) plus inflammation biomarkers (CRP and/or AGP) were measured, it would be necessary to follow the adjustment procedure for ferritin (6) or sTfR (12). However, if both ferritin and sTfR were measured, it may be advantageous to calculate TBI as it is expressed on the basis of body weight and, thus, allow for an evaluation of the full range of iron status from deficiency to excess within a population, thereby providing information on iron status beyond that of ferritin or sTfR alone (17).

TBI has been used in the United States to assess the prevalence of ID of PSC, WRA, and pregnant women (16, 17) and for monitoring purposes in the Healthy People 2020 objectives (18). There is good agreement between the estimated prevalence of ID by TBI and the previously described ferritin multiple-indicator model in PSC, WRA (16), and pregnant women (17). However, TBI has been used less often in areas with a high prevalence of inflammation and infections including malaria. Further studies are needed to examine the validity of TBI as a good marker of ID, particularly because inflammation was not accounted for during the development of TBI. The ratio of sTfR and ferritin has also been used to estimate ID (17), and further analyses should examine whether our results apply to these ratios.

In conclusion, this study, to the best of our knowledge, is the first to examine the association of TBI with inflammation and malaria across multiple settings and in both PSC and WRA. Our analysis shows that the prevalence of low TBI is underestimated if it is not adjusted by inflammation, particularly in children who are living in areas with a high prevalence of inflammation or infections.
